# Effect of screening for type 2 diabetes on risk of cardiovascular disease and mortality: a controlled trial among 139,075 individuals diagnosed with diabetes in Denmark between 2001 and 2009

**DOI:** 10.1007/s00125-017-4299-y

**Published:** 2017-08-23

**Authors:** Rebecca K. Simmons, Simon J. Griffin, Torsten Lauritzen, Annelli Sandbæk

**Affiliations:** 1 Department of Public Health, Section of General Practice, Aarhus University, Aarhus, Denmark; 2 Danish Diabetes Academy, Odense University Hospital, Odense, Denmark; 3 Aarhus Institute of Advanced Studies, Aarhus, Denmark; 4 MRC Epidemiology Unit, University of Cambridge School of Clinical Medicine, Box 285 Institute of Metabolic Science, Cambridge Biomedical Campus, Cambridge, CB2 0QQ UK; 5 Primary Care Unit, Institute of Public Health, University of Cambridge, Cambridge, UK

**Keywords:** Cardiovascular disease, General practice, Mortality, Population, Screening, Trial, Type 2 diabetes

## Abstract

**Aims/hypothesis:**

There is continuing debate about the net benefits of population screening for type 2 diabetes. We compared the risk of cardiovascular disease (CVD) and mortality among incident cases of type 2 diabetes in a screened group with those in an unscreened group.

**Methods:**

In this register-based non-randomised controlled trial, eligible individuals were all men and women aged 40–69 years without known diabetes, registered with a general practice in Denmark (*n* = 1,912,392). Between 2001 and 2006, 153,107 individuals registered with 181 practices participating in the Anglo–Danish–Dutch Study of Intensive Treatment in People with Screen-Detected Diabetes in Primary Care (ADDITION)-Denmark study were sent a diabetes-risk-score questionnaire. Individuals at moderate-to-high risk were invited to visit their family doctor for assessment of diabetes status and cardiovascular risk (screening group). The 1,759,285 individuals registered with all other practices in Denmark constituted the retrospectively constructed no-screening (control) group. In this post hoc analysis, we identified individuals from the screening and no-screening groups who were diagnosed with diabetes between 2001 and 2009 (*n* = 139,075), and compared risk of CVD and mortality in these groups between 2001 and 2012.

**Results:**

In the screening group, 27,177/153,107 (18%) individuals attended for screening, of whom 1533 were diagnosed with diabetes. Between 2001 and 2009, 13,992 people were newly diagnosed with diabetes in the screening group (including those diagnosed by screening) and 125,083 in the no-screening group. Between 2001 and 2012, the risks of CVD and mortality were lower among individuals with diabetes in the screening group compared with individuals with diabetes in the no-screening (control) group (CVD HR 0.84, 95% CI 0.80, 0.89; mortality HR 0.79, 95% CI 0.74, 0.84).

**Conclusions/interpretation:**

A single round of diabetes screening and cardiovascular risk assessment in middle-aged Danish adults in general practice was associated with a significant reduction in risk of all-cause mortality and CVD events in those diagnosed with diabetes.

**Electronic supplementary material:**

The online version of this article (doi:10.1007/s00125-017-4299-y) contains peer-reviewed but unedited supplementary material, which is available to authorised users.

## Introduction

The potential benefits of screening and early treatment for type 2 diabetes have been widely debated. Modelling studies suggest that screening might be both effective and cost-effective [1–5]. Screening and early treatment for diabetes appear to be associated with limited harms [6, 7]. Health check programmes including diabetes risk assessment have been proposed or introduced in a number of countries [8, 9]. However, trials of population-based screening for type 2 diabetes [10] and related cardiovascular risk factors [11] have failed to show significant overall benefit.

While the effect of screening at the population level might be smaller than expected, there may be benefits for those found to have diabetes. Results from the Anglo–Danish–Dutch Study of Intensive Treatment In People with Screen-Detected Diabetes in Primary Care (ADDITION)-Europe (ClinicalTrials.gov, number NCT00237549) showed that individuals diagnosed with diabetes and treated earlier had a risk of mortality that was similar to that reported for people of the same age without diabetes in the general population in Denmark [12]. One of the challenges of demonstrating potential benefit for those found to have diabetes following screening is that it would not be ethical to conduct a clinical trial of screening and early intervention compared with screening and delayed intervention [13]. As such, we cannot directly observe the magnitude of cardiovascular risk reduction that might occur among individuals with diabetes found by screening compared with those with no screening and hence no treatment until the time of clinical diagnosis [5]. Furthermore, simply comparing screen-detected individuals with clinically diagnosed individuals in a parallel cohort design tends to overestimate benefit because of lead and length time biases.

Between 2001 and 2006, a population-based cardiovascular risk assessment and diabetes screening programme was introduced in general practices in the Danish arm of the ADDITION-Europe study [14]. In the present study, using a controlled design, the Danish national registration system allows us to quantify the extent to which screening brings forward the diagnosis of diabetes, and to conduct a post hoc comparison of the risk of mortality and cardiovascular events among individuals with incident diabetes in the screening group and individuals with incident diabetes in the no-screening (control) group.

## Methods

ADDITION-Europe is a cluster-randomised trial comparing the effects of screening for type 2 diabetes followed by intensive multifactorial therapy of individuals with screen-detected diabetes and screening followed by routine care [14, 15]. We report results from a post hoc analysis using data from the screening phase of the Danish arm of the study in conjunction with data from Danish national registers. Ethical approval for the ADDITION-Denmark study was granted by a local scientific committee (number 20000183). As this was a registry-based study using anonymised data, participants did not give informed consent. This approach was approved by the Danish Data Protection Agency and the Danish Health and Medicine Authority.

### Screening programme

Full details of the programme have been reported [11, 12, 14]. In brief, we performed a population-based stepwise screening programme in people aged 40 to 69 years, without known diabetes, between 2001 and 2006 [14–16]. All general practices in five out of 16 counties in Denmark (Copenhagen, Aarhus, Ringkoebing, Ribe and South Jutland) were invited to take part in ADDITION-Denmark (*n* = 744); 209 (28.1%) accepted.

Eligible individuals registered with the 181 practices who agreed to take part were sent a diabetes-risk-score questionnaire, [15, 16] with an invitation to visit their general practitioners for a diabetes test and a cardiovascular risk assessment if they scored ≥5 (maximum 15) points or were invited when visiting the practice for another reason (*n* = 35 practices). No reminders were sent. Participants who attended a screening appointment underwent measurement of height, weight and blood pressure. A capillary blood sample was taken for testing of random blood glucose (RBG). A venous blood sample was taken for measurement of total cholesterol and HbA_1c_. General practitioners were encouraged to calculate the European Heart SCORE [17] during the appointment, to inform individuals about their score and provide appropriate advice and treatment to those at high risk. Individuals with an RBG ≥ 5.5 mmol/l or HbA_1c_ ≥ 5.8% were invited to return to the practice for a fasting blood glucose (FBG) (capillary) test. An OGTT was performed at the same consultation if FBG was 5.6–6.1 mmol/l and/or HbA_1c_ ≥ 5.8%. WHO 1999 criteria were used to diagnose diabetes [18], including the requirement for a confirmatory test on another day.

In the screening group, participants diagnosed with type 2 diabetes were subsequently managed according to the treatment regimen to which their practice was allocated: routine care or intensive treatment [12].

### Sampling frame

**Fig. 1:**
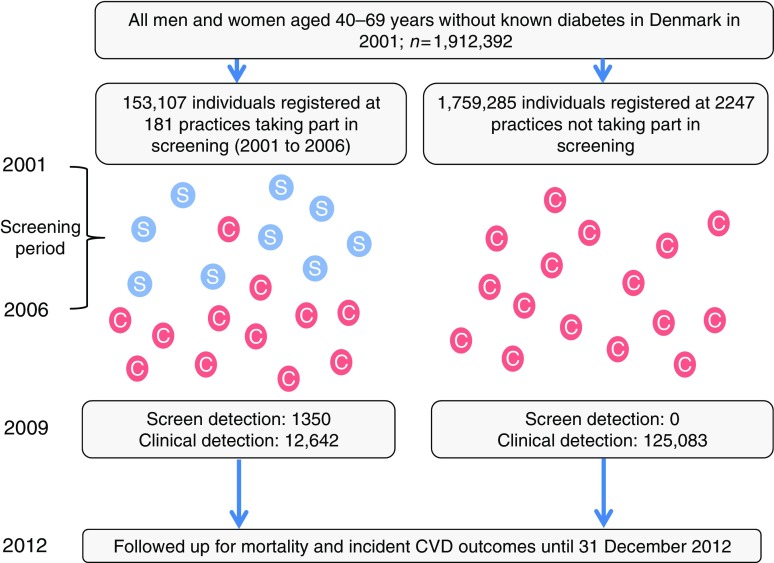
Visual representation of sampling frame. The ‘S’ in a blue circle denotes individuals detected by the ADDITION stepwise screening programme. The ‘C’ in a red circle denotes individuals with clinically diagnosed diabetes

We identified all eligible individuals in the original ADDITION-Denmark study (*n* = 153,107), including those who did not attend for screening, on the Danish National Registry system (the screening group). Using the same registry, we also identified all individuals aged 40–69 years without known diabetes who, between 2001 and 2006, were registered with general practices that were not invited to take part in ADDITION-Denmark or who declined to take part in ADDITION-Denmark (*n* = 1,759,285) (the no-screening control group). We then identified individuals from the screening and no-screening groups who were diagnosed with incident diabetes between 1 January 2001 and 31 December 2009 (Fig. 1). We included individuals diagnosed with diabetes during this period based on recent estimates of lead time, which suggest that there is around 3 years between detection by screening and clinical diagnosis. Including individuals diagnosed with diabetes in the 3 years (2006–2009) following the end of the ADDITION screening phase (2001–2006) would therefore capture most individuals in the no-screening group who could have been diagnosed by screening if they had been in the screening group. Our definition of incident diabetes for both groups was a proxy measure based on date of inclusion in the Danish National Diabetes Register [19]. We linked information about individuals diagnosed with diabetes to other Danish registers using unique civil registration numbers. We retrieved information on age, sex, education, immigration/emigration, citizenship, redeemed cardioprotective medication and chronic disease. Education was categorised according to Unesco’s International Standard Classification of Education [20]. We grouped data on citizenship into European and non-European as a proxy for ethnicity.

### Outcomes

Participants were followed for a median of 6.2 years until 31 December 2012, when national registers were searched for information on vital status and incident cardiovascular disease (CVD) events. For death, the primary outcome was all-cause mortality (based on underlying cause of death). Secondary outcomes were cardiovascular-, cancer- and diabetes-related mortality. Cause-specific deaths were coded blind to study group using ICD-10 codes (www.who.int/classifications/icd/en/; electronic supplementary material [ESM] Table 1). For CVD, the primary outcome was a composite of first event of cardiovascular death, non-fatal IHD (ICD-10 codes I20–I25, I46) or non-fatal stroke (ICD-10 code I6*). Data were gathered from the National Patient Registry, which records all inpatient and outpatient hospitalisations in Denmark.

### Statistical analysis

We summarised the characteristics of all individuals diagnosed with incident diabetes between 2001 and 2009 separately in the screening and no-screening groups. Date of entry to the study was the date of inclusion on the diabetes register. Individuals were censored on the date of first event, date of emigration or 31 December 2012, whichever was earliest. We estimated HRs comparing mortality outcomes and incident CVD in people diagnosed with diabetes in the screening and no-screening groups with a Cox proportional hazards regression model. As allocation to screening and no-screening groups was at the practice level, robust standard errors were calculated that take into account the two-level structure of the data [21]. We adjusted for age, sex, education and prevalent chronic disease (ischaemic heart disease [IHD], stroke, cancer) at baseline. To account for differences in social structure, we stratified the baseline hazards by county. We conducted a subgroup analysis, re-running the all-cause mortality model separately by age (<55 years or ≥55 years) and sex. To calculate the proportion of individuals redeeming cardioprotective medication in each calendar year, we included all individuals who were alive on 31 December of the year in question and who had previously been diagnosed with diabetes. All analyses were completed using Stata Version 14.1 (STATA, College Station, TX, USA). Statistical significance was inferred at a two-tailed *p* < 0·05.

## Results

Of 153,107 eligible people in the screening group, 27,177 (18%) attended their general practice for a diabetes test and cardiovascular risk assessment. Of these, 1533 participants (1% of those eligible for screening) were diagnosed with diabetes and agreed to take part in the study; 1406 of these were subsequently included on the diabetes register. There were 1,759,285 individuals in the no-screening group.

Between 1 January 2001 and 31 December 2009, 139,075 people from our sampling frame were diagnosed with incident diabetes and included on the Danish National Diabetes Register. Of these, 13,992 (10.1%) were in the screening group and 125,083 (89.9%) in the no-screening group. There were 83,385 (60.5%) clinically diagnosed cases of diabetes detected during the screening period (2001 to 2006) and 54,340 (39.5%) cases between 2007 and 2009. The groups were well balanced for age and citizenship (Table 1). There were slightly more men in the no-screening group (56.4%) compared with the screening group (53.6%). Compared with the no-screening group, a larger proportion of the screening group had received 15+ years of education. Slightly higher proportions of the no-screening group had experienced IHD, stroke or cancer.

**Table 1:** Characteristics of individuals with diabetes by screening group

Characteristic	Screening group *n* = 13,992	No-screening (control) group *n* = 125,083
Mean age at diagnosis (SD), years	59.9 (7.7)	59.2 (9.2)
Male sex, *n* (%)	7495 (53.6)	70,559 (56.4)
Years of education, n (%)		
0 to 10	5610 (40.1)	55,770 (44.6)
10 to 15	6237 (44.6)	55,230 (44.2)
15+	2145 (15.3)	14,083 (11.3)
European citizenship, *n* (%)^a^	13,809 (99.0)	121,572 (98.2)
Previous IHD, *n* (%)^b^	1586 (11.3)	16,217 (13.0)
Previous stroke, *n* (%)^b^	628 (4.5)	6852 (5.5)
Previous cancer, *n* (%)^b^	2027 (14.5)	19,276 (15.4)

^a^Totals do not match denominator owing to missing data

^b^Data taken from the National Patient Registry; data included from 1994 until date of diabetes diagnosis

Median diabetes duration among clinically diagnosed individuals in the screening group was 6.6 years (interquartile range [IQR] 4.6 to 9.4) compared with 8.8 years (IQR 6.9 to 10.1) in screen-detected individuals in the screening group (difference 2.2 years, *p* < 0.001).

In the first year of follow-up, there were 11,097 cases of incident diabetes. Among these individuals, larger numbers of people in the no-screening group redeemed glucose-lowering medication (49.9%) compared with clinically diagnosed (31.4%) and screen-detected individuals (20.8%) in the screening group (Table 2). This difference persisted for clinically diagnosed individuals in the screening and no-screening groups throughout follow-up. However, there was a steep increase in the proportion of screen-detected individuals redeeming glucose-lowering medication, rising from 20.8% in 2001 to 60.9% in 2009. Slightly higher numbers of clinically diagnosed individuals from the no-screening group redeemed lipid-lowering and anti-hypertensive treatment compared with clinically diagnosed individuals in the screening group throughout follow-up (difference between 3% and 5%). For individuals with screen-detected diabetes, we observed very large increases in lipid-lowering and anti-hypertensive treatment, rising from 17.5% and 51.7% in 2001, to 81.0% and 83.6% in 2009, respectively. Overall, this group redeemed the highest proportion of all cardioprotective medication by the end of follow-up.

**Table 2:** Redeemed cardioprotective medication from 2001 to 2009 by screening group

Year	Glucose-lowering medication^a^			Lipid-lowering medication^a^			Anti-hypertensive medication^a^			Total^b^
	No-screening group	Screening group		No-screening group	Screening group		No-screening group	Screening group		
		Clinically diagnosed	Screen- detected		Clinically diagnosed	Screen- detected		Clinically diagnosed	Screen- detected	
2001	4928 (49.9)	334 (31.4)	31 (20.8)	1518 (15.4)	141 (13.2)	26 (17.5)	5396 (54.6)	543 (51.0)	77 (51.7)	11,097
2002	10,028 (48.2)	641 (25.0)	106 (25.9)	4679 (22.5)	454 (17.7)	132 (32.2)	11,879 (57.1)	1289 (50.3)	250 (61.0)	23,777
2003	16,288 (49.2)	1050 (25.8)	240 (34.7)	10,703 (32.3)	1098 (27.0)	330 (47.8)	19,823 (59.9)	2183 (53.7)	459 (66.4)	37,866
2004	23,401 (50.8)	1461 (28.9)	373 (39.4)	18,936 (41.1)	1812 (35.8)	585 (61.8)	29,043 (63.0)	2913 (57.5)	664 (70.2)	52,092
2005	30,458 (52.5)	1900 (31.0)	464 (43.4)	27,176 (46.9)	2539 (41.4)	712 (67.4)	38,124 (65.8)	3707 (60.4)	787 (74.5)	65,180
2006	38,453 (54.8)	2412 (31.2)	583 (47.1)	37,091 (52.9)	3536 (45.7)	920 (74.3)	47,938 (68.3)	4854 (62.8)	948 (76.5)	79,137
2007	47,162 (56.5)	2986 (32.9)	663 (52.7)	48,218 (57.7)	4689 (51.7)	974 (77.4)	58,837 (70.4)	5967 (65.8)	1003 (79.9)	93,851
2008	57,087 (58.0)	3618 (34.3)	737 (58.5)	60,981 (62.0)	5895 (55.9)	1018 (80.8)	71,373 (72.5)	7213 (68.4)	1035 (82.1)	110,198
2009	67,071 (59.2)	4292 (36.6)	764 (60.9)	71,654 (63.3)	6763 (57.7)	1016 (81.0)	83,834 (74.0)	8273 (70.6)	1048 (83.6)	126,225

Parentheses show the proportion (%) of individuals in each screening group who redeemed a particular class of cardio-protective medication in each year

^a^Anatomical Therapeutic Chemical (ATC) codes: glucose-lowering medication (A*); lipid-lowering medication (C10*); anti-hypertensive medication (CO7*, CO8*, C09*)

^b^Based on all individuals alive on 31 December of the year in question who had previously been diagnosed with diabetes

**Fig. 2:**
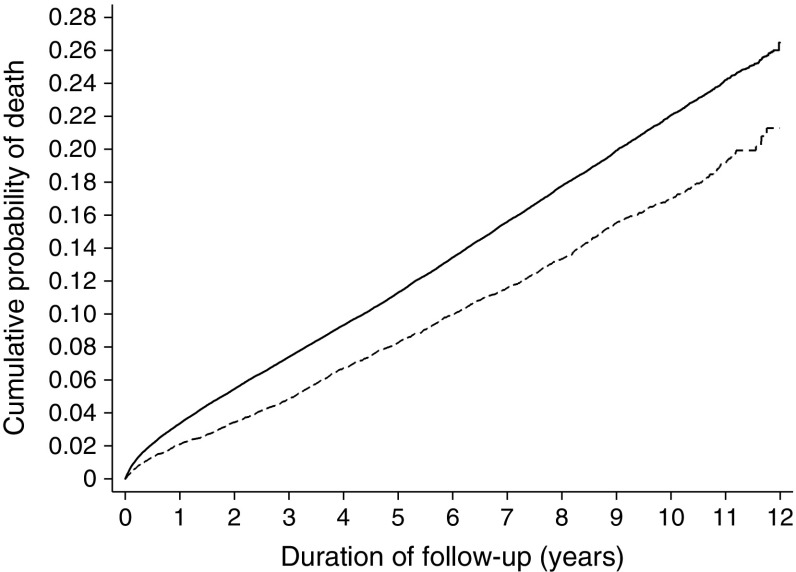
Cumulative incidence of all-cause mortality in individuals with diabetes in the screening and no-screening groups (2001 to 2012). The model is unadjusted. Solid line, no-screening (control) group; dashed line, screening group

Median duration of follow-up was 6.2 years (IQR 4.2 to 8.8), with 898,285 person-years of observation. During follow-up, there were 1775 deaths in people with diabetes in the screening group (12.7%) and 19,739 deaths in people with diabetes in the no-screening group (15.8%) (Table 3). The HR for mortality was 0.79, 95% CI 0.74, 0.84, *p* < 0.0001 (Fig. 2). The most common cause of death was cancer (*n* = 10,500; 38.2%). In sub-group analyses, the HR for mortality was similar among individuals aged <55 years and ≥55 years (HR 0.81, 95% CI 0.71, 0.93, and HR 0.78, 95% CI 0.73, 0.84, respectively). The HRs for mortality were 0.82 (95% CI 0.76, 0.88) in men and 0.75 (95% CI 0.68, 0.82) in women.

**Table 3:** Incidence of all-cause, cardiovascular-, cancer- and diabetes-related mortality, and CVD events, in individuals with diabetes, by screening group (2001 to 2012)

Variable	Screening group (*n* = 13,992)			No-screening group (*n* = 125,083)			Crude HR (95% CI)	Adjusted HR^a^ (95% CI)
	Number of events	Person-years follow-up	Rate per 1000 person-years (95% CI)	Number of events	Person-years follow-up	Rate per 1000 person-years (95% CI)		
All-cause mortality	1775	97,265	18.2 (17.4, 19.1)	19,739	801,019	24.6 (24.3, 25.0)	0.71 (0.68, 0.75)	0.79 (0.74, 0.84)
Cardiovascular mortality	509	97,265	5.2 (4.8, 5.7)	5835	801,019	7.3 (7.1, 7.5)	0.70 (0.64, 0.77)	0.80 (0.72, 0.88)
Cancer mortality	954	97,265	9.8 (9.2, 10.5)	9546	801,019	11.9 (11.7, 12.2)	0.79 (0.74, 0.84)	0.83 (0.77, 0.89)
Diabetes-related mortality	121	97,265	1.2 (1.0, 1.5)	1701	801,019	2.1 (2.0, 2.2)	0.57 (0.48, 0.69)	0.66 (0.54, 0.81)
Composite cardiovascular event (first of CVD death, non-fatal IHD or non-fatal stroke)	2854	86,317	33.1 (31.9, 34.3)	28,487	698,893	40.8 (40.3, 41.2)	0.78 (0.75, 0.81)	0.84 (0.80, 0.89)

^a^HRs were estimated with a Cox proportional hazards regression model. Robust standard errors were calculated that take into account the two-level structure of the data and any potential correlation between individuals within practices

Models were adjusted for age, sex, education and prevalent chronic disease (IHD, stroke, cancer); baseline hazards were stratified by county

Cardiovascular mortality and cancer mortality were both significantly lower in the screening group compared with the no-screening group (HR 0.80, 95% CI 0.72, 0.88 and HR 0.83, 95% CI 0.77, 0.89, respectively). Diabetes was listed anywhere on the death certificate in 1822 individuals (121 in the screening group and 1701 in no-screening group), with a significant difference between groups in diabetes-related mortality (HR 0.66, 95% CI 0.54, 0.81).

**Fig. 3:**
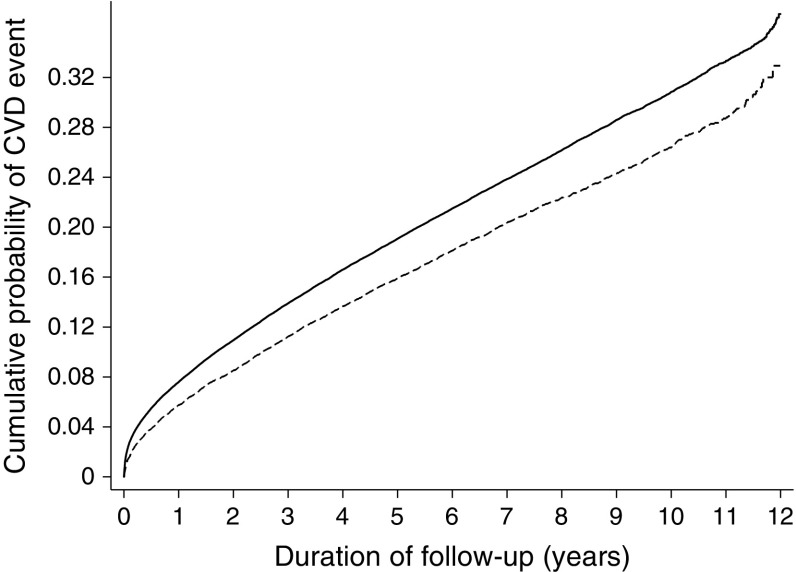
Cumulative incidence of a composite CVD event among individuals with diabetes in the screening and no-screening groups (2001 to 2012). The model is unadjusted. Solid line, no-screening (control) group; dashed line, screening group

There were 2854 first CVD events among people with diabetes in the screening group (20.4%) and 28,487 first CVD events among people with diabetes in the no-screening group (22.8%) (Table 3), with a significant difference between groups (HR 0.84, 95% CI 0.80, 0.89) (Fig. 3).

## Discussion

In this very large population-based sample of middle-aged Danish adults with 898,285 person-years of follow-up, a single round of diabetes screening and cardiovascular risk assessment was associated with a 21% reduction in all-cause mortality rate and a 16% reduction in CVD events between 2001 and 2012 in individuals diagnosed with diabetes between 2001 and 2009. Individuals with clinically diagnosed diabetes were identified on average 2.2 years later than individuals with diabetes detected by screening.

One argument for considering screening for type 2 diabetes is the historical observation [22] that there is an extended latent or pre-clinical phase (lead time) in which people could be diagnosed and during which earlier treatment might have a beneficial long-term effect. More recently, changes in clinical practice with greater testing and public awareness have probably led to a shortening of this latent period. Data from the parallel-group population-based Ely study suggested that the lead time is relatively short at 3.3 years [23]. This is comparable with our more contemporary estimate of 2.2 years. However, the lead time may be longer in less developed health systems and/or in more deprived populations. In addition, the historical estimate of 9 to 12 years by Harris et al. [22] and the more recent estimate of 6 years from Porta et al. [24] relates to the true point of onset of diabetes. This is not the same as the point at which diabetes is detectable by screening, especially if screening is infrequent and not 100% sensitive. The period between true onset and clinical diagnosis of diabetes may be long precisely because there are few clinical manifestations during this period [23].

Even with a relatively modest lead time, our screening programme was associated with a significant reduction in mortality and incident CVD in individuals with diabetes over 6 years of follow-up. As only 10.1% of individuals in the screening group were actually diagnosed by screening, it is likely that the programme had wider effects in this cohort e.g. by delaying diagnosis and providing lifestyle advice (and perhaps treatment) among those screened and found to be at risk who were later diagnosed clinically. The difference in mortality and CVD might also have been driven by screening practices that were vigilant for diabetes even after the programme had finished, contributing to continued earlier detection and the higher diabetes incidence observed in screening compared with no-screening practices.

Our findings support results from modelling studies showing that screening programmes could contribute to a reduction in risk of mortality and cardiovascular morbidity in screen-detected individuals [1–5]. Herman et al. used a validated computer simulation model in ADDITION-Europe to show that screening and routine care, compared with a 3 year delay in diagnosis and routine care, was associated with a 17% relative risk reduction in all-cause mortality after 5 years [5]. They argue that the benefits of screening and treatment primarily accrue from early diagnosis and by hastening the treatment of CVD risk factors in the lead time [5]. We observed a rapid increase in the proportion of screen-detected individuals who redeemed cardioprotective medication during follow-up. However, larger proportions of clinically diagnosed individuals in the no-screening group redeemed medication compared with clinically diagnosed individuals in the screening group. As individuals in the no-screening group were diagnosed at a later stage in the disease trajectory, they may have had higher cholesterol, blood glucose and blood pressure values at diagnosis compared with the screening group, necessitating higher levels of cardioprotective medication. It is likely that promotion of healthy behaviour change also impacted on CVD and mortality rates in the screening group. Those who attended screening reported their smoking status at baseline (28%), which was similar to national self-reported smoking prevalence data in 2004 (Danish National Health Service survey) [25]. One-third of screen-detected individuals in ADDITION-Denmark reported that they had stopped smoking at 5 year follow-up. Furthermore, this cohort lost an average of 2 kg in weight [12]. If similar behavioural responses were observed among other individuals diagnosed with diabetes in the screening group, this suggests potential mechanisms for the risk reduction observed other than prescribed treatment. We also observed lower rates of cancer incidence in the screening group, which might be linked to changes in health behaviour and prescribing [26].

In a separate paper [11], we examine the impact of the ADDITION-Denmark screening programme at the population level, e.g. comparing all individuals aged 40 to 69 years in the screening and no-screening groups, and showed no long-term reduction in mortality or CVD. As such, our results mirror those from trials of screening for other conditions, which have shown reductions in disease-specific mortality but not in overall mortality [27]. There appeared to be beneficial effects for all those diagnosed with diabetes in the screening practices, regardless of the mode of diagnosis e.g. by screen detection or by clinical diagnosis. However, this benefit is too small to impact on overall population risk of CVD events and mortality [10, 11].

### Strengths and limitations

This very large controlled trial with long-term follow-up included all individuals aged 40 to 69 years diagnosed with diabetes in Denmark between 2001 and 2009. Outcome ascertainment was robust. The National Death Registry estimates 100% coverage of mortality based on death certificates. All-cause mortality is an all-inclusive measure that addresses both direct and indirect effects of screening, and puts disease-specific mortality reduction in the context of other competing risks [27]. We were able to ascertain which individuals were living in Denmark in 2001 and censor those who emigrated during follow-up. Deaths and CVD events were coded blind to study group.

Our definition of clinically diagnosed diabetes was a proxy measure based on date of inclusion in the Danish National Diabetes Register, where individuals are classified as having diabetes according to a number of criteria [19, 28]. Using registry-defined diabetes ensures that the entire Danish population is covered by uniform inclusion criteria and the dropout rate is nil. However, we did not have formal clinical diagnosis of diabetes or the date of diagnosis. A recent validation of the algorithm for including individuals in the Register suggests that it has a sensitivity ≥95% and a positive predictive value of around 80% [19]. The same report also suggests that around 20% of diabetes diagnoses in the Register may represent false-positive inclusions of people with frequent measurements of blood glucose who do not have diabetes. This may help account for the higher incidence of diabetes in the screening group and help explain the lower levels of cardioprotective medication redeemed by clinically diagnosed individuals in the screening group. Many high-risk individuals would have undergone frequent measurements of blood glucose during the screening programme, delineating them with a diabetes diagnosis on the Register, when they did not in fact have diabetes and were therefore unlikely to receive cardioprotective treatment.

In total, 1406/1533 (92%) of individuals diagnosed with screen-detected diabetes in ADDITION-Denmark were added to the Diabetes Register with a median delay of 56 days (R. K. Simmons, unpublished data). While this proxy date means our estimate of diabetes duration is probably shorter than the actual length, this is unlikely to be differential by group. By only including individuals aged 40 to 69 years in our study, we assume the number of clinically diagnosed type 1 diabetes cases is likely to be low and similar in both groups.

A limitation of our study is the non-randomised design; we cannot eliminate the possibility of selection bias and residual confounding. Groups were well balanced for most characteristics at baseline. However, our findings might have been influenced by the higher levels of education and the slightly lower levels of pre-existing chronic disease in the screening group. We did adjust for age, sex, education and prevalent chronic disease, which had a small impact on the effect size. Including adjustment by county had a large impact, reducing the effect size considerably, though the hazard remained significant. It is likely that adjusting for county took account of some of the potential socioeconomic differences across different regions in Denmark.

We tried to minimise lead and length time biases by comparing outcomes for all individuals diagnosed with diabetes in the screening and no-screening groups. Further, we extended the inclusion period to 3 years beyond the end of the formal screening programme so that people who could have been detected by screening (had they been in the screening group) were included. However, the small difference in the overall incidence between the groups suggests that some of the observed effect may be due to residual lead and length time bias. Participation in the programme may also have impacted on subsequent diabetes detection rates in screening practices.

While we were able to compare trends in redeemed cardioprotective medication to explore a potential mechanism for the observed difference in outcomes, we did not have population-level data on dietary, physical activity or smoking behaviour. The majority of participants were white, the main ethnic group in Denmark, which also limits generalisability to other settings.

In conclusion, a single round of diabetes screening and cardiovascular risk assessment in middle-aged Danish adults performed in general practice was associated with a 21% reduction in all-cause mortality rates and a 16% reduction in CVD events between 2001 and 2012 among people diagnosed with diabetes between 2001 and 2009. Screening resulted in cases being identified, on average, 2.2 years earlier.

## Electronic supplementary material

ESM Table 1 (PDF 10 kb)

## Abbreviations

ADDITION: Anglo–Danish–Dutch Study of Intensive Treatment in People with Screen-Detected Diabetes in Primary Care
CVD: Cardiovascular disease
FBG: Fasting blood glucose
IHD: Ischaemic heart disease
RBG: Random blood glucose
