# Supplementary material for: Effect of screening for type 2 diabetes on risk of cardiovascular disease and mortality: a controlled trial among 139,075 individuals diagnosed with diabetes in Denmark between 2001 and 2009
Source: Diabetologia. 2017 Aug 23;60(11):2192–9. doi: 10.1007/s00125-017-4299-y (PMC6108415; doi:10.1007/s00125-017-4299-y)
Supplement: Supplementary file 1 — (PDF 10 kb) [file 125_2017_4299_MOESM1_ESM.pdf]

Electronic supplementary material

Table 1 Cause-specific mortality groupings by ICD-10 code

**Cause-specific mortality**	**ICD-10 code used**
Cardiovascular	F01*, I*
Cancer	C*, D0* to D4*,
Suicide / violence / accident	F1*, V*, W*, X*, Y*
Other	A*, B*, D5* to D8*, E*, F00*, F02* to F09*, F2* to F9*, G*, H*, J*, K*, L*, M*, N*, P*, Q*
Unknown	R*
**Diabetes-related mortality**	E10* to E14*
